# Metabolomics comparison of serum and urine in dairy cattle using proton nuclear magnetic resonance spectroscopy

**DOI:** 10.5713/ab.20.0870

**Published:** 2021-04-23

**Authors:** Jun Sik Eom, Eun Tae Kim, Hyun Sang Kim, You Young Choi, Shin Ja Lee, Sang Suk Lee, Seon Ho Kim, Sung Sill Lee

**Affiliations:** 1 Division of Applied Life Science (BK21), Gyeongsang National University, Jinju 52828, Korea; 2 National Institute of Animal Science, Rural Development Administration, Cheonan 31000, Korea; 3 Institute of Agriculture and Life Science & University-Centered Labs, Gyeongsang National University, Jinju 52828, Korea; 4 Ruminant Nutrition and Anaerobe Laboratory, College of Bio-industry Science, Sunchon National University, Suncheon 57922, Korea

**Keywords:** Dairy Cattle, ^1^H-NMR Spectroscopy, Metabolites, Serum, Urine

## Abstract

**Objective:**

The aim of the study was to conduct metabolic profiling of dairy cattle serum and urine using proton nuclear magnetic resonance (^1^H-NMR) spectroscopy and to compare the results obtained with those of other dairy cattle herds worldwide so as to provide a basic dataset to facilitate research on metabolites in serum and urine.

**Methods:**

Six dairy cattle were used in this study; all animals were fed the same diet, which was composed of total mixed ration; the fed amounts were based on voluntary intake. Blood from the jugular neck vein of each steer was collected at the same time using a separate serum tube. Urine samples were collected by hand sweeping the perineum. The metabolites were determined by ^1^H-NMR spectroscopy, and the obtained data were statistically analyzed by performing principal component analysis, partial least squares-discriminant analysis, variable importance in projection scores, and metabolic pathway data using Metaboanalyst 4.0.

**Results:**

The total number of metabolites in the serum and urine was measured to be 115 and 193, respectively, of which 47 and 81, respectively were quantified. Lactate (classified as an organic acid) and urea (classified as an aliphatic acylic compound) exhibited the highest concentrations in serum and urine, respectively. Some metabolites that have been associated with diseases such as ketosis, bovine respiratory disease, and metritis, and metabolites associated with heat stress were also found in the serum and urine samples.

**Conclusion:**

The metabolites measured in the serum and urine could potentially be used to detect diseases and heat stress in dairy cattle. The results could also be useful for metabolomic research on the serum and urine of ruminants in Korea.

## INTRODUCTION

Metabolomics is a new biological analysis method that provides precise analysis of molecules (molecular weight of >1,000). It is typically used to investigate the activity and status of cellular and organismal metabolism on a global or network scale to delineate the end point of physiology and pathophysiology [1–3]. Analytical technologies that are used in metabolomics include nuclear magnetic resonance (NMR) spectroscopy, gas chromatography-mass spectrometry (GC-MS), and liquid chromatography-mass spectrometry (LC-MS) [4,5]. The NMR spectroscopy has a lower metabolite coverage than GC-MS and LC-MS; however, it has a short analysis time, low cost, and is easily comparable worldwide [6]. For this reason, research on metabolites using NMR spectroscopy as well as MS instruments (GC-MS and LC-MS) is actively being conducted.

Representative metabolomics research using serum and plasma are performed to obtain diagnostic or therapeutic biomarkers of human diseases (Alzheimer’s, cancer, etc.) [7–9], or to identify potential biomarkers that may improve muscle and meat quality traits (water-holding capacity, meat color, tenderness, flavor, palatability, etc.) in animals [10]. Representative metabolomic studies using urine have been conducted for the detection of steroids biomarkers (screening for cancer, mental and central nervous system disorders, and endocrine diseases; monitoring of drug therapy; doping control, etc.) [11].

In recent years, research on metabolomics using biofluids (rumen fluid, serum, plasma, milk, urine, feces, etc.) of ruminants has been actively conducted worldwide. Metabolomics research using ruminant serum and plasma have been conducted to investigate the relationship between negative energy balance (NEB) and early lactation in dairy cows [12], feed efficiency in black angus steers [13], heat stress (HS) in beef cattle breeds [14], and in search of biomarkers of ketosis and bovine respiratory diseases (BRD) [15,16]. Research on metabolomics using ruminant urine has focused on urea metabolism and recycling [17], postpartum diseases such as metritis and lameness in dairy cows [18,19], HS in beef cattle breeds [14], and nitrogen efficiency in dairy cows [20]. Such studies have helped in the diagnosis and prevention of metabolic diseases and increased the productivity of ruminants.

In Korea, metabolomics research using ruminant biofluids has been conducted for the comparison of volatile fatty acid and monosaccharide metabolite concentrations in the rumen fluid of Hanwoo cattle using proton NMR (^1^H-NMR) spectroscopy, high-performance liquid chromatography, and high-performance anion-exchange chromatography [21]. In recent years, metabolomic comparisons of various Hanwoo cattle biofluids (rumen fluid, serum, and urine) have also been conducted using ^1^H-NMR spectroscopy [22]. In addition, research on the metabolite changes in the milk and feces of dairy cattle with different feeding ratios of roughage and concentrate [23,24], and metabolomic comparison of dairy cattle rumen fluid and milk using ^1^H-NMR spectroscopy have been performed [25]. However, overall, metabolomics research using humans, foods, and monogastric animals are aplenty, while studies on metabolomics using biofluids of ruminants are nascent. Therefore, more research using ruminant biofluids should be conducted.

In this study, we measured the metabolites in dairy cattle serum and urine by using ^1^H-NMR spectroscopy. The metabolites were then identified and classified to construct a database of each sample, wherein the concentration of each metabolite was provided. In addition, the roles of the metabolites observed in this study were compared to those reported in a previous study. The results of this study provide useful a database for the analysis of metabolites in ruminant serum and urine in Korea.

## MATERIALS AND METHODS

All experimental protocols used in this study were approved by the National Institute of Animal Science, Department of Animal Resources Development, Dairy Science Division (Cheonan, Chungcheongnam-do, Korea; NIAS-201908).

### Animals and sample collection

Six dairy cattle (48.09±18.60 months-old; body weight, 558.83±43.28 kg; parity, 1.33±0.82; milk yield, 27.82±4.72 kg/d) were used in this study. All animals were fed the same diet, which was composed of total mixed ration (TMR); the amounts fed were based on voluntary intake. The chemical composition of the TMR is presented in Table 1. The contents of dry matter (Method 934.01), crude protein (Method 976.05), calcium (Method 927.02), and phosphorus (Method 3964.06) in TMR was assayed as described by AOAC [26,27]. The contents of neutral detergent fiber and acid detergent fiber in TMR was assayed as described by Van Soest et al [28]. Blood samples were collected after feeding, from the jugular neck vein of each animal was collection at the same time using separate serum tubes. The blood samples were centrifuged at 806×g and 4°C for 15 min, and aliquots of the upper layer (serum) were stored at −80°C for later ^1^H-NMR spectroscopy analysis. Urine samples were collected by hand sweeping the perineum, thus stimulating each dairy cattle to urinate, and immediately stored at −80°C for later ^1^H-NMR spectroscopy analysis.

### ^1^H-NMR spectroscopy analysis

Saline buffer in NaCl concentration of 0.9% weight/volume in 100% deuterium oxide (D_2_O) solvent was prepared. The stored serum samples were centrifuged at 14,000×g and 4°C for 10 min. The supernatant 200 μL and 400 μL of saline buffer was added to the 5 mm NMR tube for ^1^H-NMR spectroscopy spectral analysis [29].

Urine samples were added to 0.2 M sodium phosphate buffer (pH 7.0). The samples were centrifuged at 14,000×g and 4°C for 10 min and collected 400 μL supernatant. Supernatant was added to 230 μL of buffer and was measured of pH 7.0±0.1. The mixture solution (630 μL) was added to 2 mM standard buffer solution (TSP; 2,2,3,3-d4-3(Trimethylsilyl)propionic acid sodium salt) 60 μL and TSP concentration in the total solution was adjusted to 0.2 mM [20]. The prepared sample was transferred to 5 mm NMR tube for ^1^H-NMR spectroscopy spectral analysis.

The spectra of serum and urine samples were obtained on a SPE-800 MHz NMR-MS Spectrometer (Bruker BioSpin AG, Fällanden, Switzerland) at 298 K using a 5 mm triple-resonance inverse cryoprobe with Z-gradients (Bruker BioSpin CO., Billerica, MA, USA). The pulse sequence used for the serum and urine was a Carr-Purcell-Meiboom-Gill pulse sequence and NOESY presaturation collecting 64,000 data points with 128 transients, a spectral width of 16,025.641 Hz, a relaxion delay of 4.0 s, and an acquisition time of 2.0 s [30].

### ^1^H-NMR spectroscopy data identification, quantification, and statistical analysis

The processed spectra were imported the Chenomx NMR suite 8.4 software (Chenomx Inc, Edmonton, AB, Canada) for identification and quantification. The baseline and phase were matched for comparison between samples using the Chenomx processor. The following procedure was employed for qualitative and quantitative analysis of the metabolites in samples. The spectral width was 10 ppm and was referenced to the TSP signal at 0 ppm. The resources used were the Livestock Metabolite Database (http://www.lmdb.ca), Bovine Metabolite Database (http://www.bmdb.ca), and Chenomx library manager. Metabolite qualitative and quantitative were performed using the Chenomx profiler program.

Statistical analyses of the metabolite data were conducted using Metaboanalyst version 4.0 (http://www.metaboanalyst.ca), an open source R-based program for metabolomics. The resulting metabolites were subjected to sample normalization by “sum”, data transformation by “log”, and data scaling by “pareto” during statistical analysis. Univariate Student’s t-tests were used to quantify difference between metabolite profiles of the biofluid samples. Principal component analysis (PCA) and partial least square-discriminant analysis (PLS-DA) were used as multivariate data analysis techniques to generate a classification model and provide quantitative information for discriminating the metabolites. The different biofluid metabolites were determined on the basis of a statistically significant threshold of variable importance in projection (VIP) scores. Metabolites with VIP scores higher than were obtained 1.5 were obtained through PLS-DA.

Metabolic pathways analysis was performed using a *Bos taurus* pathway library. Metabolic pathways were measured, different metabolites in biofluid metabolites of the other studied animals were statistically analyzed by Metaboananlyst 4.0 for metabolic pathways analysis, which is based on database source by Kyoto encyclopedia of genes and genomes (http://www.kegg.com).

## RESULTS

### Metabolites measured and quantified via ^1^H-NMR spectroscopy

**Figure 1:**
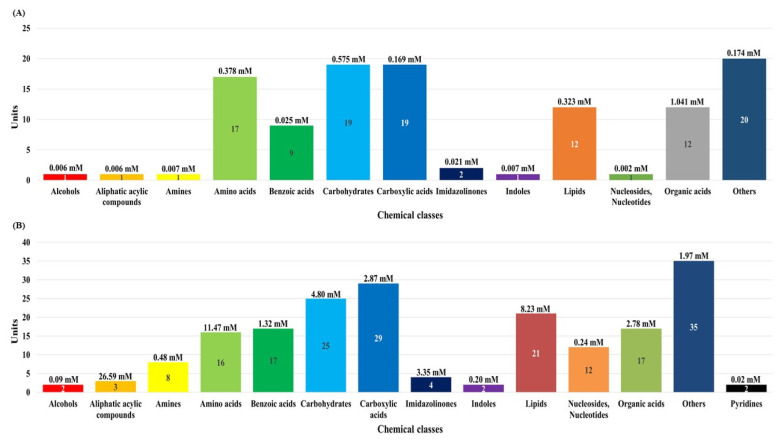
The classification of measured metabolites according to chemical class in serum (A) and urine (B) by proton nuclear magnetic resonance spectroscopy analysis. Each square box color indicates the classification of metabolites, the numbers represents the measured metabolites, and the numbers in parentheses indicate the sum of the total concentrations of the measured metabolites.

In this study, although the number of dairy cattle used the small, simultaneous metabolic profiling of different serum and urine were conducted to foster future research. The results in Figure 1 and Supplementary Tables S1, S2, and S3 reveal the measured and quantified compounds in serum (A) and urine (B), which were measured by using ^1^H-NMR spectroscopy. In the serum, 115 metabolites were measured and categorized into 13 chemical classes. The classes with the most metabolites were other (20), carbohydrates (19), and carboxylic acids (19); the classes with the highest concentrations were organic acids (1.041 mM), carbohydrates (0.575 mM), and amino acids (0.378 mM). In addition, 47 metabolites were quantified (n≥4) in the serum. In the urine, 193 metabolites were measured and categorized into 14 chemical classes. The classes with the most metabolites were other (35), carboxylic acids (29), and carbohydrates (25); the classes with the highest concentrations were aliphatic acylic compounds (26.59 mM), amino acids (11.47 mM), and lipids (8.23 mM). Additionally, 81 metabolites were quantified (n≥4) in the urine.

### ^1^H-NMR spectra and statistical analysis

**Figure 2:**
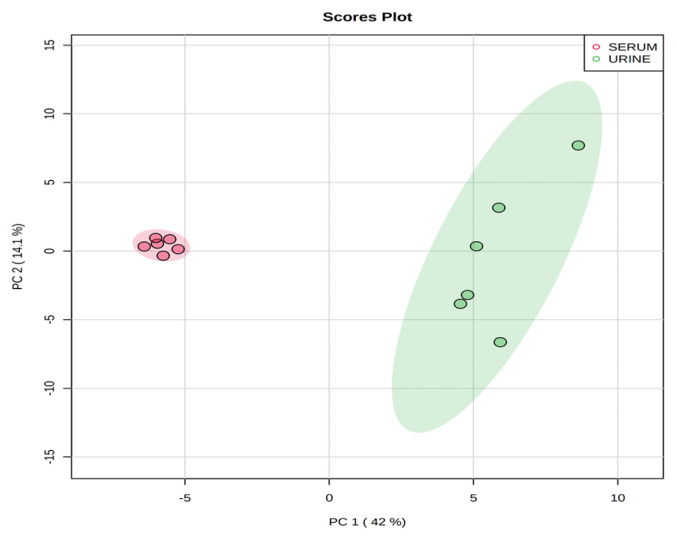
Principal components analysis score plot based on metabolites data in serum and urine by proton nuclear magnetic resonance spectroscopy analysis. On the score plot, each point represents an individual sample, with the red dot representing the serum group (n = 6), and the green dot representing the urine group (n = 6). The abscissa and ordinate represent the variance associated with PC 1 and 2, respectively.

**Figure 3:**
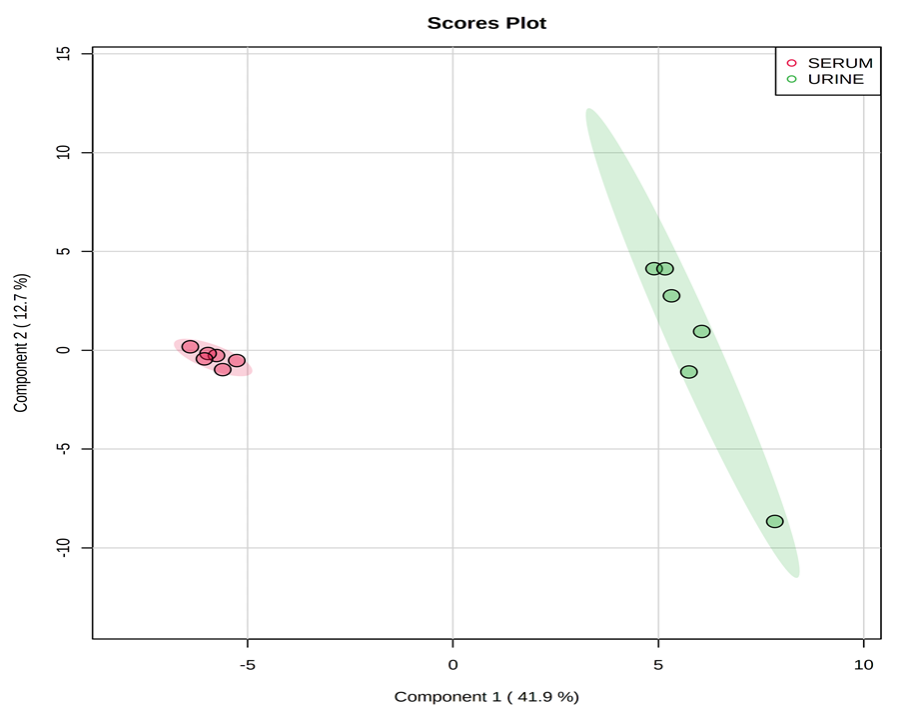
Partial least square-discriminant analysis score plot of serum and urine by proton nuclear magnetic resonance spectroscopy analysis. The shaded ellipses represent the 95% confidence interval estimated from the score. On the score plot, each point represents an individual sample, with the red dot representing the serum group (n = 6), and the green dot representing the urine group (n = 6). The abscissa and ordinate represent the variance associated with components 1 and 2, respectively.

**Figure 4:**
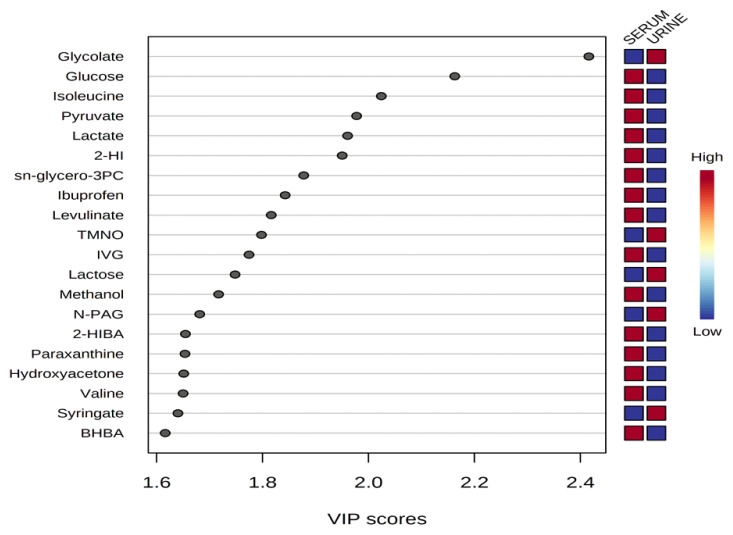
Variable importance in projection (VIP) scores of metabolites in serum and urine by proton nuclear magnetic resonance spectroscopy analysis. The selected metabolites were those with VIP score >1.5. Heat map with red or blue boxes on the right indicates high and low abundance ratio, respectively, of the corresponding metabolite in serum and urine. The VIP score was based on the partial least square-discriminant analysis model. Metabolite abbreviation: 2-HI, 2-hydroxyisovalerate; sn-glycero-3PC, sn-glycero-3-phosphocholine; TMNO, Trimethylamine *N*-oxide; IVG, *N*-isovaleroyglycine; N-PAG, *N*-phenylacetylglycine; 2-HIBA, 2-hydroxyisobutyrate; paraxanthine, 1,7-Dimethylxanthine; BHBA, 3-hydroxybutyrate. VIP score value: glycolate, 2.4159; glucose, 2.1628; isoleucine, 2.0242; pyruvate, 1.9776; lactate, 1.9605; 2-HI, 1.9503; sn-glycero-3PC, 1.8777; ibuprofen, 1.8427; levulinate, 1.8165; TMNO, 1.798; IVG, 1.7744; lactose, 1.7481; methanol, 1.7171; N-PAG, 1.6814; 2-HIBA, 1.6545; paraxanthine, 1.6536; hydroxyacetone, 1.6512; valine, 1.65; syringate, 1.6402; BHBA, 1.6163.

Representative ^1^H-NMR spectra of 40 and 57 metabolites measured as single, doublet, and triplet peaks in the serum and urine samples (as well as the reference substance TSP) are shown in Supplementary Figures S1 and S2, respectively. Our data showed that there were differences in the metabolites between the serum and urine. To visualize the differences among the metabolite data, we performed PCA and PLS-DA (Figures 2 and 3). Both score plots revealed differences in the biofluids, which were explained by PC 1 (42%) and PC 2 (14.1%) in the PCA and component 1 (41.9%) and component 2 (12.7%) in the PLS-DA. These results indicated significant variation among the different classes and concentrations of metabolites in the two biofluids. As shown in Figure 4, the two biofluids exhibited completely different metabolite profiles. The VIP scores were also utilized to quantify the metabolites that affected the difference (VIP score >1.5) between the two biofluids (Figure 4). In the serum, 15 metabolites (glucose, isoleucine, pyruvate, lactate, 2-hydroxyisovalerate, sn-glycero-3-phosphocholine, ibuprofen, levulinate, N-isovaleroyglycine, methanol, 2-hydroxyisobutyrate, paraxanthine, hydroxyacetone, valine, and 3-hydroxybutyrate [BHBA]) were present in significantly higher concentrations than in the urine. In the urine, five metabolites (glycolate, trimethylamine *N*-oxide, lactose, *N*-phenylacetylglycine, and syringate) were present in significantly higher concentrations than in the serum.

### Top 30 average concentrations of serum and urine metabolites

The top 30 average concentrations of metabolites in the serum and urine are shown in Tables 2 and 3, respectively. Among the metabolites quantified (n≥4) in the serum, lactate, acetate (classified as an organic acid), and glucose (classified as a carbohydrate) had the highest concentrations. In contrast, erythritol (classified as a carbohydrate), glutaric acid monomethyl ester (classified as a lipid), and acetoacetate (classified as a carbohydrate) had the lowest concentrations. Among the metabolites quantified (n≥4) in the urine, urea (classified as an aliphatic acylic compound), hippurate (classified as an amino acid), and glycolate (classified as a lipid) had the highest concentrations. In contrast, 3-hydroxykynurenine (classified as an organic acid), 3-indoxylsulfate (classified as an indole), and acetylsalicylate (classified as a benzoic acid) had the lowest concentrations.

### Common metabolites and metabolic pathways in the serum and urine

**Figure 5:**
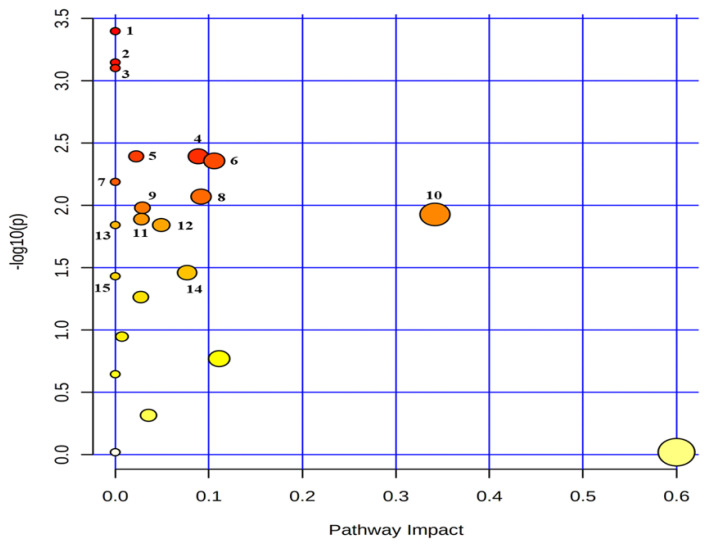
Metabolic pathway mapping of common quantified (n≥4) metabolites between serum and urine. The pathway impact analysis was performed using Metaboanalyst 4.0 software. The x-axis represents the pathway impact, and y-axis represents the pathway enrichment. The results are presented graphically as a bubble plot. The darker color and larger size represent higher p-value from enrichment analysis and greater impact from the pathway topology analysis, respectively. Metabolic pathway name: 1, phenylalanine metabolism; 2, alanine, aspartate and glutamate metabolism; 3. aminoacyl-tRNA biosynthesis; 4, glutathione metabolism; 5, primary bile acid biosynthesis; 6, glyoxylate and dicarboxylate metabolism; 7, selenocompound metabolism; 8, pyruvate metabolism; 9, glycolysis/gluconeogenesis; 10, glycine, serine and threonine metabolism; 11, porphyrin and chlorophyll metabolism; 12, histidine metabolism; 13, beta-alanine metabolism; 14, citrate cycle (tricarboxylic acid cycle); 15, propanoate metabolism.

Twenty-seven common metabolites were quantified (n≥4) in the serum and urine (Supplementary Table S1–3). An analysis showed that the common metabolites (Table 4, Figure 5) followed 15 metabolic pathways, among which the top five pathways were phenylalanine metabolism; alanine, aspartate and glutamate metabolism; aminoacyl-tRNA biosynthesis; glutathione metabolism; and primary bile acid biosynthesis. The metabolites in the relevant pathways were mainly amino acids.

## DISCUSSION

Ketosis is a metabolic disease in lactating dairy cattle and is, typically caused by high milk production or extreme peripartum reduction in energy intake and is specifically a high risk for cows suffering from severe NEB [31,32]. Diseases like this negatively impact the health, reproductive, performance, milk production capacity, and milk composition of cows, leading to a decrease in dairy industry profitability [32]. A typical method for the diagnosis of ketosis in lactating dairy cattle is to measure the BHBA concentration associated with ketone body metabolites (BHBA, acetoacetate, and acetone) in the blood (serum and plasma) and urine [32]. Moreover, a high concentration ketone body metabolites in blood and urine have been associated with decreased feed intake and increased other periparturient diseases [32]. In this study, ketone body metabolites were measured in serum and urine. According to Xu et al [12], changes in the concentrations of acetone, arginine, BHBA, glucose, glycine, kynurenine, and panthothenate in the plasma may be an indicator of NEB. Further, as reported by Luke et al [15], an increase in the BHBA concentrations in early lactation dairy cattle was positively correlated with acetate, betaine, creatine, glycine, and phosphocholine, and negatively correlated with alanine, dimethyl sulfone, glucose, lactate, and valine in the serum. All metabolites associated with NEB were measured in this study, except for kynurenine; further, the metabolites associated with increased BHBA concentration in dairy cattle during early lactation were also measured in this study, except for dimethyl sulfone and phosphocholine. Therefore, metabolites measured by serum ^1^H-NMR spectroscopy could potentially be used to diagnose and, perhaps, prevent ketosis, which could significantly affect dairy industry profitability.

The BRD is a multifactorial disease that can significantly impact the economic prosperity and welfare of the farm industry [16]. Diseases like this, caused by a complex of physiological and environmental stressors, precede farm admittance; for example, transportation, mixing with unfamiliar animals/herds, or exposure to viral microbial population agents can cause BRD [33]. According to Blakebrough-Hall et al [16], animals suffering from BRD had higher concentrations of alpha-glucose, acetone, BHBA, creatine, creatinine, ethanol, hydroxybutyrate, isobutyrate, isoleucine, isopropanol, leucine, mannose, phenylalanine, and pyruvate metabolites. In contrast, 1-methylhistidine, acetate, alanine, citrate, glucose, glutamine, glycine, glycoprotein acetyl, hydroxyisobutyrate, low-density lipoprotein (LDL), tyrosine, and valine metabolite were found in lower concentrations. In this study, BRD-associated metabolites were measured, except for alpha-glucose, citrate, ethanol, glutamine, glycoprotein acetyl, hydroxybutyrate, hydroxyisobutyrate, isobutyrate, isopropanol, LDL, phenylalanine, and tyrosine in the serum. Hence, metabolites measured in the serum in this study supplement the literature regarding the understanding of BRD-associated metabolites and could possibly be used to verify the occurrence of BRD in cattle.

The HS has become a major issue due to the acceleration of global warming. Exposure to HS, results in reduced milk production and quality in ruminants and makes them vulnerable to diseases [34]. Such consequences could damage the livestock industry. Liao et al [14] conducted a comparative study of metabolite changes in the blood and urine of three cattle breeds exposed to HS. In Xuanhan yellow cattle (XHC), glucose, lactate, and pyruvate associated with glycolysis and aconitate, citrate, and fumarate associated with the tricarboxylic acid (TCA) cycle were found in higher concentrations in the serum and urine [14]. In Simmental×Xuanhan yellow crossbred cattle (SXC), asparagine, creatinine, glutamate, glutamine, ornithine, and urea associated with the amino acid metabolism, and aconitate, citrate, and fumarate associated with the TCA cycle were observed in higher concentrations. Finally, Jersey×Maiwa yak crossbred cattle (JMY), asparagine, creatinine, fumarate, glutamine, methionine, ornithine, phenylalanine, pyruvate, tyrosine, and urea associated with amino acid metabolism were found in higher concentration in the serum [14]. Higher concentrations of aconitate, citrate, and succinate were found in the urine of the XHC and SXC breeds, higher concentrations of methylcitrate and methylmalonate (associated with TCA cycle) were found in the urine of the JMY breed [14]. In this study, glycolysis was measured in relation to the metabolites in the serum. In addition, pyruvate and creatinine were measured as metabolites associated with amino acid metabolism, and succinate was measured as a metabolite associated with the TCA cycle in the serum. In addition, all metabolites associated with the TCA cycle were measured in this study, except for methylcitrate and methylmalonate. Hence, the metabolites measured in this study could potentially be used to verify the occurrence of HS in cattle.

Metritis is a uterine inflammation that occurs during the first three weeks post-parturition; it not only increases veterinary costs but also damages the livestock industry [19,35] as a result of lower reproductive efficiency, increased culling rates, and decreased milk production [19]. According to Dervishi et al [19], using urine from lactating dairy cattle, the candidate group of metabolites for the diagnosis of metritis consists of increased concentrations of 1,3-dihydroxyacetone, 3-aminoisobutyrate, acetylsalicylate, ascorbate, betaine, cysteine, galactose, glutamine, glycolate, guanidoacetate, hypoxanthine, lysine, N-acetylaspartate, O-phosphocholine, threonine, xylose, trans-aconitate, and π-methylhistidine metabolite and, decreased concentrations of uracil and urea metabolites. In this study, acetylsalicylate, betaine, galactose, glycolate, guanidoacetate, O-phosphocholine, urea, xylose, trans-aconitate, and π-methylhistidine metabolites were measured in the urine. Therefore, metabolites measured in the urine in this study could be used to verify the occurrence of metritis.

## IMPLICATIONS

Proton nuclear magnetic resonance spectroscopy and statistical analyses were employed to analyze the metabolites in dairy cattle serum and urine. The metabolites measured in the serum and urine were mostly consistent with those reported in studies conducted abroad, and may be useful for predicting diseases (ketosis, bovine respiratory disease, and metritis) and heat stress in dairy cattle. Furthermore, this report on metabolites in ruminant serum and urine will contribute to future ruminal metabolism studies in Korea.

## Figures and Tables

**Table 1:** Ingredients and nutrients of the experimental diets

Items	Value (% of dry matter)
Ingredients (% of dry matter)	
Concentrate	15.3
Soybean meal	2.40
Corn silage	47.2
Alfalfa hay	7.10
Tall fescue	9.40
Timothy	5.90
Energy booster^1)^	7.10
Cash gold^1)^	4.50
Lyzin-Plus^2)^	0.20
Limestone^3)^	0.20
Zin care^1)^	0.10
Supex-F^1)^	0.50
Trace minerals^4)^	0.05
Vitamins premix^5)^	0.05
Chemical composition (% of dry matter basis)	
Dry matter (%)	53.2
Crude protein	10.0
Neutral detergent fiber	28.2
Acid detergent fiber	16.9
Calcium	0.40
Phosphorus	0.15

1) Cofavet, Cheonan, Korea. Zin Care, contained 16 GDU/g protease bromelain, 2.0×10^8^ cfu/g. Supex-F, Contained 99% protected fat from of palm oil.

2) A.N.Tech, Cheonan, Korea. Lyzin-Plus, contained 6.0% Zn, 0.9% Cu, 1.4% Mn, 5.0% chelated glycine.

3) Sungshin minefield, Jeongseon, Korea.

4) Trace minerals, contained 0.40% Mg, 0.20% K, 4.00% S, 0.08% Na, 0.03% Cl, 400 mg of Fe/kg, 60,042 mg of Zn/kg, 16,125 mg of Cu/kg, and 42,375 mg of Mn/kg.

5) Vitamins premix, provided approximately 5,000 KIU of vitamin A/kg, 1,000 KIU of vitamin D/kg, 33,500 mg of vitamin E/kg, and 2,400 mg of vitamin C/kg.

**Table 2:** Average concentrations (mean±standard deviation) of top 30 metabolites in serum by proton nuclear magnetic resonance spectroscopy analysis (n≥4)

Metabolites	Classification	Concentration (μM)
Lactate	Organic acid	588.12±152.69
Acetate	Organic acid	307.77±59.89
Glucose	Carbohydrate	263.62±44.85
3-hydroxybutyrate	Lipid	150.02±50.44
Glycine	Amino acid	91.37±18.98
2-hydroxyisovalerate	Lipid	55.20±11.10
Malate	Organic acid	48.84±8.34
Alanine	Amino acid	47.50±12.63
Gluconate	Organic acid	45.56±18.61
Creatine	Amino acid	36.45±18.93
Methanol	Alcohol	23.44±1.74
Pyruvate	Carbohydrate	23.28±11.12
3-methylglutarate	Lipid	22.98±9.06
Acetone	Other	22.97±24.12
Lactose	Carbohydrate	19.10±9.19
Leucine	Amino acid	17.68±9.98
Lactulose	Carbohydrate	15.98±4.70
Isoleucine	Amino acid	15.40±7.24
Glycylproline	Carboxylic acid	14.00±1.81
Formate	Organic acid	13.88±1.64
3-hydroxyisovalerate	Carboxylic acid	12.38±5.91
sn-glycero-3-phosphocholine	Other	12.12±4.85
Valine	Amino acid	12.12±6.67
Galactarate	Other	11.74±2.76
Levulinate	Other	11.32±5.08
Betaine	Other	10.50±5.41
Phenylacetate	Organic acid	9.90±2.13
Erythritol	Carbohydrate	9.60±1.25
Glutaric acid monomethyl ester	Lipid	9.22±2.46
Acetoacetate	Carbohydrate	9.08±5.54

**Table 3:** Average concentrations (mean±standard deviation) of top 30 metabolites in urine by proton nuclear magnetic resonance spectroscopy analysis (n≥4)

Metabolites	Classification	Concentration (μM)
Urea	Aliphatic acylic compound	24,962.90±7,653.27
Hippurate	Amino acid	7,812.64±2,148.48
Glycolate	Lipid	5,889.98±4,997.68
Allantoin	Imidazolinone	2,671.04±2,028.96
Trimethylamine *N*-oxide	Aliphatic acylic compound	1,629.08±1,209.95
Creatine	Amino acid	1,265.60±1291.76
Acetate	Carbohydrate	1,201.68±617.70
*N*-phenylacetylglycine	Amino acid	1,128.52±683.98
Glycylproline	Carboxylic acid	430.73±145.07
Creatine phosphate	Carboxylic acid	412.50±670.93
Malate	Organic acid	235.22±96.43
Sebacate	Lipid	225.05±93.98
Fructose	Carbohydrate	214.30±149.05
Salicylate	Organic acid	210.08±263.92
Acetoacetate	Carbohydrate	204.02±141.22
3-methylglutarate	Lipid	201.05±62.55
Tryptophan	Amino acid	198.78±51.08
Syringate	Benzoic acid	197.92±241.18
Glycine	Amino acid	191.63±143.21
Homocystine	Carboxylic acid	183.43±89.95
3-phenylpropionate	Other	172.50±149.71
Gentisate	Benzoic acid	165.53±124.72
Biotin	Other	140.36±121.05
Vanillate	Benzoic acid	135.02±87.41
Xanthine	Nucleoside, nucleotide	134.16±34.93
Galactitol	Carbohydrate	123.06±135.36
4-hydroxyphenylacetate	Benzoic acid	122.65±44.93
Acetylsalicylate	Benzoic acid	120.98±125.86
3-indoxylsulfate	Indole	120.42±51.15
3-hydroxykynurenine	Organic acid	119.93±66.03

**Table 4:** Pathway analysis with common quantified (n≥4) metabolites in serum and urine

Metabolic pathway	Total Cmpd^1)^	Hits^2)^	p-value	−Log (p-value)	FDR^3)^	Impact^4)^
Phenylalanine metabolism	12	2	4.00×10^−4^	3.40	6.06×10^−3^	0.00
Alanine, aspartate and glutamate metabolism	28	2	7.13×10^−4^	3.15	6.06×10^−3^	0.00
Aminoacyl-tRNA biosynthesis	48	2	7.91×10^−4^	3.10	6.06×10^−3^	0.00
Glutathione metabolism	28	1	4.04×10^−3^	2.39	1.69×10^−2^	0.09
Primary bile acid biosynthesis	46	1	4.04×10^−3^	2.39	1.69×10^−2^	0.02
Glyoxylate and dicarboxylate metabolism	32	4	4.40×10^−3^	2.36	1.69×10^−2^	0.11
Selenocompound metabolism	20	1	6.48×10^−3^	2.19	2.13×10^−2^	0.00
Pyruvate metabolism	22	2	8.50×10^−3^	2.70	2.44×10^−2^	0.09
Glycolysis/gluconeogenesis	26	1	1.05×10^−2^	1.98	2.54×10^−2^	0.03
Glycine, serine and threonine metabolism	34	5	1.18×10^−2^	1.93	2.54×10^−2^	0.34
Porphyrin and chlorophyll metabolism	30	2	1.29×10^−2^	1.89	2.54×10^−2^	0.03
Histidine metabolism	16	1	1.44×10^−2^	1.84	2.54×10^−2^	0.05
beta-Alanine metabolism	21	1	1.44×10^−2^	1.84	2.54×10^−2^	0.00
Citrate cycle (tricarboxylic acid cycle)	20	2	3.46×10^−2^	1.46	5.86×10^−2^	0.08
Propanoate metabolism	23	1	3.70×10^−2^	1.43	5.86×10^−2^	0.00

1) Total Cmpd, the total number of compounds in the pathway.

2) Hit, the actually matched number from the user uploaded data.

3) FDR, the p-value adjusted using false discovery rate.

4) Impact, the pathway impact value calculated from pathway topology analysis.
