# Supplementary material for: Metabolomics comparison of serum and urine in dairy cattle using proton nuclear magnetic resonance spectroscopy
Source: Anim Biosci. 2021 Apr 23;34(12):1930–9. doi: 10.5713/ab.20.0870 (PMC8563233; doi:10.5713/ab.20.0870)
Supplement: Supplementary file 1 [file ab-20-0870-suppl.pdf]

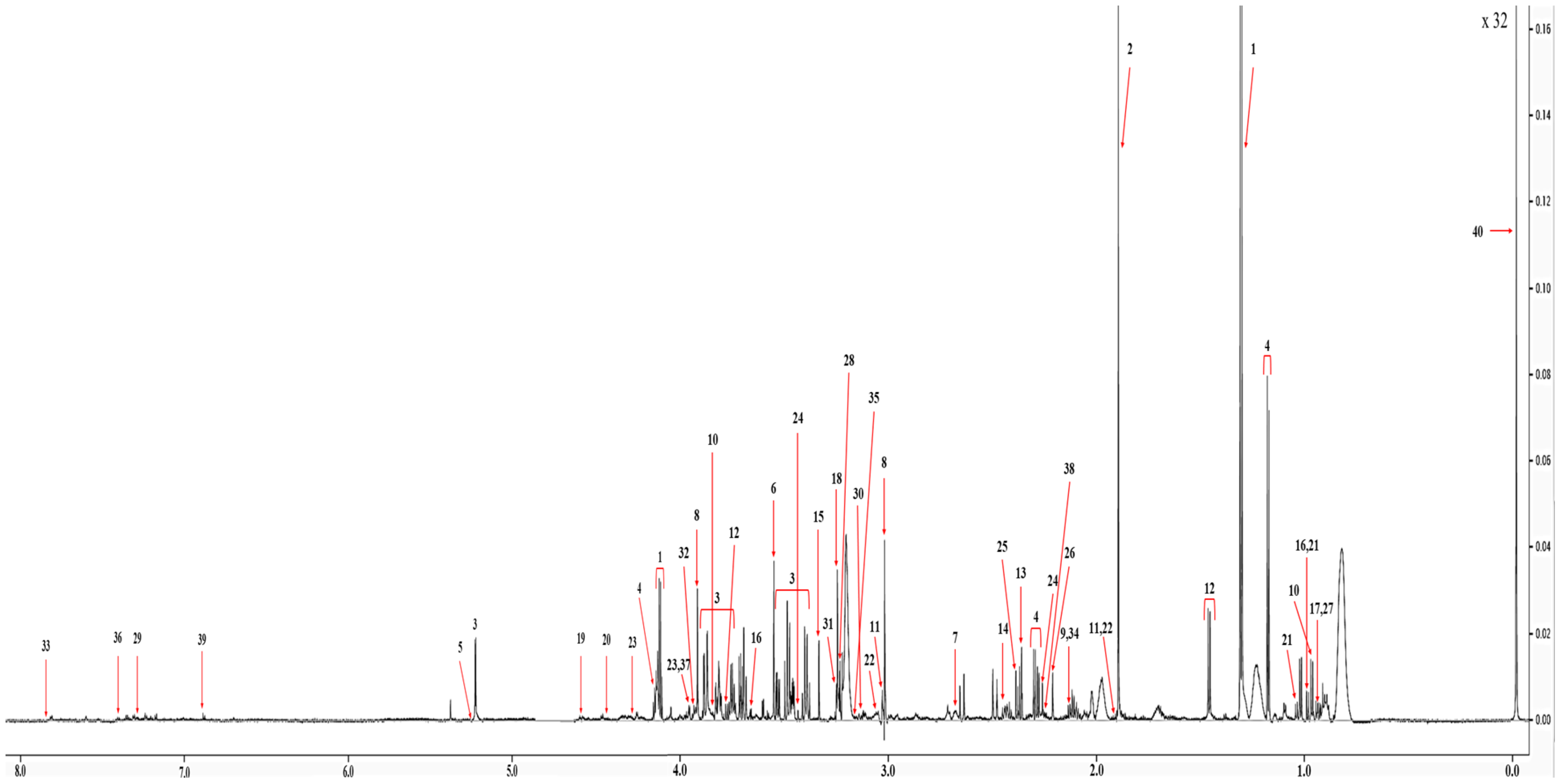

Supplementary Figure 1. Representative 800 MHz proton nuclear magnetic resonance spectra (0.0 ~ 10.0 ppm) of serum samples.

1. Lactate, 2. Acetate, 3. Glucose, 4. 3-hydroxybutyrate, 5. Ribose, 6. Glycine, 7. Malate, 8. Creatine, 9. Glutamine, 10. 2-hydroxyisovalerate, 11. Lysine, 12. Alanine, 13. Pyruvate, 14. 3-hydroxy-3-methylglutarate, 15. Methanol, 16. Isoleucine, 17. 3-methylglutarate, 18. Trimethylamine *N*-oxide, 19. Glutathione, 20. Lactose, 21. Valine, 22. Omithine, 23. Galactarate, 24. Acetoacetate, 25. Succinate, 26. Acetone, 27. Leucine, 28. Carnitine, 29. Phenylacetate, 30. Malonate, 31. Betaine, 32. Creatine phosphate, 33. 1,7-dimethylxanthine, 34. O-acetylcholine, 35. *N*-nitrosodimethylamine, 36. *N*-phenylacetylglycine, 37. Glycolate, 38. p-cresol, 39. 4-hydroxy-3-methoxymandelate, 40. TSP.

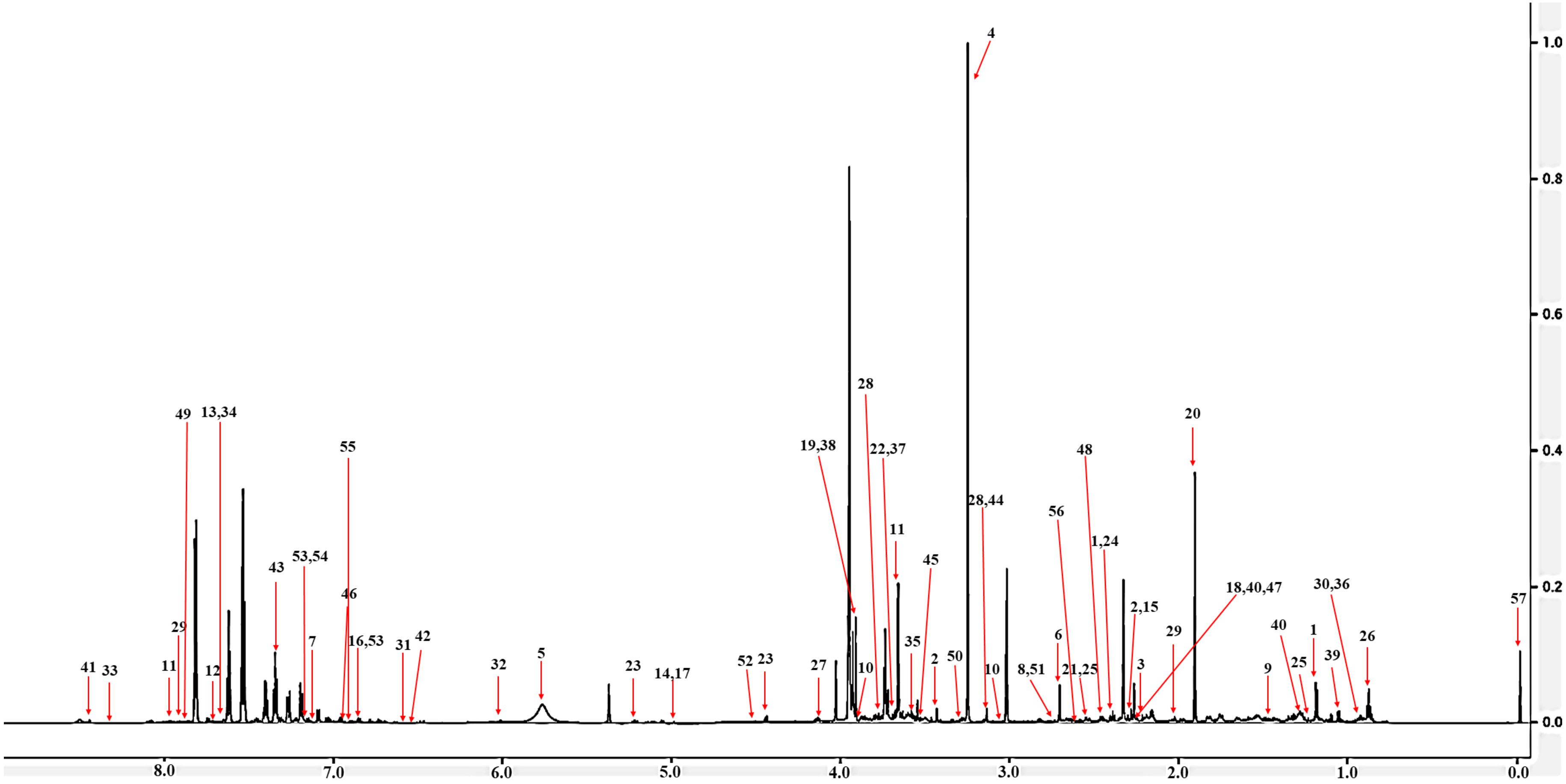

Supplementary Figure 2. Representative 800 MHz proton nuclear magnetic resonance spectra (0.0 ~ 10.0 ppm) of urine samples.

1. 3-hydroxybutyrate, 2. Acetoacetate, 3. Acetone 4. Trimethylamine *N*-oxide, 5. Urea 6. Dimethylamine, 7. Histamine 8. Sarcosine, 9. Alanine 10 creatine, 11 *N*-phenylacetylglycine, 12 tryptophan, 14. 3-hydroxymandelate, 15. Acetylsalicylate, 16 Gentisate 17. Mandelate 18. p-cresol, 19. syringate, 20. Acetate 21. Citrate 22. Galactitol 23. Lactose 24. Succinate 25. 3-hydroxyisovalerate 26. 3-methyl-2-oxovalerate, 27. 5-aminolevulinate 28. Malonate 29. *N*-acetyltyrisube, 30. Pantothenate, 31. trans-aconitate, 32. Allantoin, 33. Imidazole, 34. 3-Indoxylsulfate 35. 5-hydroxyindole-3-acetate, 36. 3-Methylglutarate, 37, Ethylene glycol, 38. Glycolate 39. Methylsuccinate, 40. Sebacate, 41. Formate, 42. Fumarate, 43. Nicotinurate, 44. *N*-nitrosodimethylamine 45. Phenylacetate 46. Salicylate, 47 Succinylacetone, 48. 3-phenylpropionate 49. 4-pyridoxate 50. Betaine, 51. Biotin 52. Cellobiose, 53. Desaminotyrosine, 54. Indole-3-acetate, 55. Kynurenate, 56. Riboflavin, 57. TSP.

Supplementary Table 1. Measured metabolites concentration in serum and urine samples by proton nuclear magnetic resonance spectroscopy analysis (mean ± standard deviation, n≥2)

Metabolites (μM)	Serum	Urine	Metabolites (μM)	Serum	Urine
Alcohols			***Benzoic acids***		
Methanol	23.44 ± 1.74	Not detected (ND)	5-methoxysalicylate	ND	47.23 ± 36.58
Aliphatic acylic compounds			Acetylsalicylate	ND	120.98 ± 125.86
O-phosphocholine	ND	1.50 ± 0.36	Gallate	ND	83.73 ± 30.97
*Trimethylamine *N*-oxide	6.33 ± 4.06	1629.08 ± 1209.95	Gentisate	ND	165.53 ± 124.72
Urea	ND	24962.90 ± 7653.27	Homogentisate	2.90 ± 0.00	71.15 ± 27.93
Amines			Mandelate	ND	48.40 ± 34.16
Carnosine		27.48 ± 14.00	o-cresol	ND	96.00 ± 63.23
Dimethylamine		75.03 ± 35.86	p-cresol	ND	43.60 ± 25.64
Histamine	ND	20.10 ± 9.84	Salicylurate	ND	93.10 ± 15.81
Kynurenine		104.20 ± 3.39	*Syringate	1.10 ± 0.55	197.92 ± 241.18
Sarcosine		17.75 ± 7.29	Vanillate	ND	135.02 ± 87.41
Amino acids			***Carbohydrates***		
2-furoylglycine	ND	24.04 ± 13.01	*Acetoacetate	9.08 ± 5.54	204.02 ± 141.22
*Alanine	47.50 ± 12.63	76.16 ± 37.15	Citrate	ND	202.17 ± 20.72
*Anserine	7.85 ± 3.40	29.34 ± 35.55	Erythritol	9.60 ± 1.25	163.30 ± 155.14
Arginine	39.00 ± 31.54	ND	Fructose	14.40 ± 2.07	214.30 ± 149.05
*Creatine	36.45 ± 18.93	1265.60 ± 1291.76	Galactitol	ND	123.06 ± 135.36
*Glycine	91.37 ± 18.98	191.63 ± 143.21	Galactonate	ND	74.20 ± 94.47
Hippurate	ND	7812.64 ± 2148.48	Galactose	23.10 ± 0.14	129.57 ± 89.31
Histidine	ND	33.88 ± 19.68	Glucose	263.62 ± 44.85	ND
Isoleucine	15.40 ± 7.24	ND	Glucose-6-phosphate	22.13 ± 8.20	ND
Leucine	17.68 ± 9.98	ND	Glucuronate	ND	81.25 ± 17.89
Methionine	1.80 ± 0.42	ND	Glutathione	11.27 ± 1.46	ND
N-isovaleroylglycine	3.35 ± 1.21	ND	Isocitrate	ND	407.90 ± 118.65
**N*-phenylacetylglycine	6.84 ± 2.97	1128.52 ± 683.98	Lactose	19.10 ± 9.19	1295.74 ± 954.80
Tryptophan	ND	198.78 ± 51.08	Lactulose	15.98 ± 4.70	185.95 ± 86.34
Valine	12.12 ± 6.67	ND	Maltose	ND	91.10 ± 89.24
Xanthurenate	ND	38.15 ± 32.18	Mannose	15.10 ± 1.41	ND
π-Methylhistidine	ND	14.27 ± 8.11	*N*-acetylglucosamine	10.85 ± 5.16	17.70 ± 5.80
Benzoic acids			Pyruvate	23.28 ± 11.12	ND
3-hydroxymandelate	ND	19.30 ± 19.69	Ribose	82.90 ± 13.28	417.90 ± 229.95
4-hydroxy-3-methoxymandelate	2.43 ± 0.47	17.30 ± 10.13	*Succinate	7.53 ± 4.32	29.35 ± 15.62
4-hydroxyphenylacetate	ND	122.65 ± 44.93	Sucrose	4.35 ± 0.21	25.63 ± 16.43

Supplementary Table 2. *Continued*

Metabolites (μM)	Serum	Urine	Metabolites (μM)	Serum	Urine
Carbohydrates			***Lipids***		
Trehalose	ND	23.17 ± 29.98	2-hydroxyisovalerate	55.20 ± 11.10	ND
Xylose	ND	56.55 ± 7.57	2-hydroxyvalerate	ND	406.65 ± 446.82
Carboxylic acids			2-methylglutarate	ND	87.70 ± 48.18
2-hydroxyisobutyrate	1.00 ± 0.26	ND	3-hydroxy-3-methylglutarate	19.63 ± 14.67	30.95 ± 15.34
3-cholorotyosine	ND	65.30 ± 35.07	3-hydroxybutyrate	150.02 ± 50.44	266.76 ± 132.37
3-hydroxyisobutyrate	ND	19.75 ± 7.57	*3-methylglutarate	22.98 ± 9.06	201.05 ± 62.55
*3-hydroxyisovalerate	12.38 ± 5.91	22.02 ± 9.71	*Carnitine	5.75 ± 4.76	7.83 ± 3.16
3-hydroxyphenylacetate	ND	84.80 ± 35.06	Choline	ND	1.77 ± 0.38
3-methyl-2-oxovalerate	ND	219.20 ± 31.48	Ethylene glycol	ND	45.10 ± 63.54
*5-aminolevulinate	6.23 ± 3.27	25.38 ± 23.22	*Glutaric acid monomethyl ester	9.22 ± 2.46	18.20 ± 14.74
Alloisoleucine	7.15 ± 0.49	ND	Glycocholate	ND	41.38 ± 45.38
*Creatine phosphate	6.30 ± 4.62	412.50 ± 670.93	Glycolate	2.70 ± 0.99	5889.98 ± 4997.68
*Glycylproline	14.00 ± 1.81	430.73 ± 145.07	Methylsuccinate	ND	78.73 ± 76.22
*Guanidoacetate	7.33 ± 3.87	40.73 ± 25.46	Sebacate	ND	225.05 ± 93.98
Homocitrulline	ND	70.50 ± 72.69	Thymol	2.57 ± 0.47	44.83 ± 15.40
Homocystine	ND	183.43 ± 89.95	***Nucleosides, Nucleotides***		
Homovanillate	ND	25.93 ± 9.62	dTTP		16.20 ± 20.55
Hydroxyacetone	2.97 ± 0.64	ND	NADH	ND	13.80 ± 5.52
*Malonate	7.92 ± 2.49	110.72 ± 185.11	Xanthine		134.16 ± 34.93
*N*6-acetyllysine	ND	49.78 ± 11.99	***Organic acids***		
N-acetyltyrosine	ND	60.10 ± 57.45	2-hydroxybutyrate	ND	177.20 ± 168.86
N,*N*-dimethylformamide	ND	8.40 ± 0.57	3-hydroxykynurenine	ND	119.93 ± 66.03
*Pantothenate	3.48 ± 0.96	58.62 ± 36.03	*Acetate	307.77 ± 59.89	1201.68 ± 617.70
trans-aconitate	ND	12.38 ± 8.55	Butyrate	3.65 ± 1.06	ND
Imidazolinones			*Formate	13.88 ± 1.64	106.95 ± 24.43
Allantoin	ND	2671.04 ± 2028.96	Fumarate	ND	2.93 ± 1.00
Creatinine	8.65 ± 8.41	639.70 ± 530.51	Gluconate	45.56 ± 18.61	213.00 ± 193.46
Imidazole	ND	12.13 ± 4.35	Lactate	588.12 ± 152.69	ND
Urocanate	ND	23.67 ± 7.12	*Malate	48.84 ± 8.34	325.22 ± 96.43
Indoles			Nicotinurate	ND	9.83 ± 2.83
3-indoxylsulfate	ND	120.42 ± 51.15	*N*-nitrosodimethylamine	5.07 ± 3.43	70.08 ± 49.78
5-hydroxyindole-3-acetate	3.47 ± 0.31	75.16 ± 26.93	O-acetylcholine	4.10 ± 3.06	ND
Lipids			*Phenylacetate	9.90 ± 2.13	86.70 ± 97.97
2-hydroxy-3-methylvalerate	ND	427.60 ± 230.30	Salicylate	ND	210.08 ± 263.92

Supplementary Table 3. *Continued*

Metabolites (μM)	Serum	Urine	Metabolites (μM)	Serum	Urine
Organic acids			***Others***		
Succinylacetone	5.40 ± 3.39	81.48 ± 53.99	Dimethyl sulfone	ND	32.87 ± 30.86
Others			Epicatechin	ND	17.88 ± 16.86
1,6-anhydro-β-D-glucose	ND	44.85 ± 0.35	*Galactarate	11.74 ± 2.76	78.65 ± 48.36
1,7-dimethylxanthine	4.85 ± 1.03	ND	Ibuprofen	6.00 ± 2.58	ND
2-hydroxyphenylacetate	ND	40.07 ± 18.60	Indole-3-acetate	ND	86.46 ± 49.43
3-methylxanthine	1.83 ± 0.57	8.65 ± 7.42	Indole-3-lactate	ND	85.88 ± 26.19
3-phenylpropionate	ND	172.50 ± 149.71	Kynurenate	ND	9.68 ± 7.63
4-pyridoxate	ND	13.48 ± 4.71	Levulinate	11.32 ± 5.08	ND
Acetoin	ND	39.60 ± 12.59	*Melatonin	3.25 ± 0.54	64.66 ± 50.86
*Acetone	22.97 ± 24.12	63.44 ± 96.82	*N*-acetylserotonin	ND	55.45 ± 34.01
Arabinose	44.10 ± 3.45	92.40 ± 43.89	*N*-methylhydantoin	1.85 ± 0.54	22.33 ± 27.85
*Betaine	10.50 ± 5.41	33.46 ± 52.18	Pyridoxine	1.80 ± 0.29	86.65 ± 67.25
Biotin	ND	140.36 ± 121.05	Riboflavin	ND	15.10 ± 11.10
Caffeine	ND	11.52 ± 9.21	sn-glycero-3-phosphocholine	12.12 ± 4.85	ND
Cellobiose	ND	86.24 ± 69.82	Theophylline	ND	8.07 ± 2.63
Desaminotyrosine	ND	151.43 ± 46.51	τ-Methylhistidine	ND	42.40 ± 29.85

ND, Not detected

*Common metabolites were quantified (n≥4) in the serum and urine

Highlighted area quantified (n≥4) metabolites in the serum

Highlighted area quantified (n≥4) metabolites in urine
